# Children with severe disabilities: adaptation, virtual education, and prospects. Experiences of three Peruvian mothers, COVID-19 context

**DOI:** 10.25122/jml-2021-0330

**Published:** 2022-01

**Authors:** Pilar Maria Gamarra Choque, Edith Gissela Rivera Arellano, Enaidy Reynosa Navarro, Juan Méndez Vergaray, Yolanda Josefina Huayta-Franco, Melissa Fatima Muñante Toledo

**Affiliations:** 1. Postgraduate School, César Vallejo University, Lima, Peru; 2. Science and Technology Research Institute, César Vallejo University, Trujillo, Peru; 3. Faculty of Engineering and Management, National Technological University of South Lima, Lima, Peru

**Keywords:** disability, mother, distance education, COVID-19

## Abstract

This study aimed to reveal and investigate mothers’ experiences of students with severe disabilities regarding learning in distance education in Lima-Peru. This is a phenomenological study focused on understanding the world of mothers regarding the education of their children with severe disabilities. Their discourse focused on four categories: being the mother of a child with severe disability, pandemic category, virtual education, and family prospects. The participants were three mothers of children with Down Syndrome, Autism Spectrum Disorder, and Cerebral Palsy. An in-depth interview structured in 26 questions was used, applied face to face. With distance education, the mothers consider that their children’s abilities and skills have assumed a leading role, developed creativity, and employed various strategies to comply with school activities. In addition, it also strengthened their family ties despite the pandemic.

## Introduction

Diagnosing a child with a disability generates an emotional and complex impact that is difficult for parents in particular and the family, in general, to assimilate [1]. This situation is evidenced in four phases: (a) denial is characterized by the presence of ignorance and disbelief of parents, who want to hide their child’s disability by feeling self-conscious and intimidated; (b) wandering, the presence of injustice, anxiety, fear, sadness, shock, disappointment, depression, and guilt, it is considered the most stressful phase that occurs in parents; (c) despair: the parents feel exhausted and helpless when observing that there are no changes, nor improvement in the disability of their children; (d) parents accept that they have done everything possible to improve their child’s condition and found no improvement despite all their efforts, overcoming the emotions they initially felt [2, 3]. In addition, they needed emotional support [4]. Despite this, they could find emotional stability amid the pandemic. However, they were frustrated by educating their children with disabilities, aware that this implies a family commitment to guarantee their care, attention, and quality of life.

Children with disabilities are considered vulnerable and depend on specific services and trained people to develop their capacities, especially in this pandemic context, where confinement has generated anxiety and frustration [5], not only for them but also for the family. It also affects the family [6]. Faced with this context, families face a new challenge to balance the time for their work and the care of their children with disabilities from home [7] and learn the use of technologies to access virtual reality education from home [8]. Despite this, many families transformed moments of social isolation into spaces of familiar proximity, although restricted in their homes [9].

Faced with this health crisis that affected many sectors, the educational field used virtuality to develop distance education or online learning, forcing millions of teachers to use appropriate technology to teach their virtual classes for their students at home [10]. In this sense, the Peruvian State implemented the free access educational strategy “I learn at home”, aligned with the national curriculum so that children can continue learning from their homes, using various communication channels. The wide deployment of this strategy implied that teachers, students, and parents were involved in the training process. This change in the educational scenario generated an additional burden on parents who now supported the role of teachers and, at the same time, shared responsibilities in their work and household chores [11].

Non-face-to-face education reduces the learning opportunities of students with disabilities because they lose the quality of particular attention that they regularly need [12]. Therefore, this educational process demands activities that involve eye contact, physical, personalized attention, and interpersonal stimulation [13]. This situation also represents a significant challenge for the parents of students with disabilities since the need to provide additional attention to their child significantly impacted the quality of life of the family members, affecting employment, income, and finances [14]. In addition, during this health crisis, these parents have been more vulnerable to stressful situations due to fears related to the pandemic and the great responsibility involved in raising children with disabilities, affecting interaction with them [15].

However, in the face of all measures adopted for education in general, many students with disabilities did not have access to the services offered by special education during the pandemic [16]. Consequently, the research objective of our study was to analyze and reflect on the role of mothers in the educational process of their children with severe disabilities during the COVID-19 context in Peru.

## Material and Methods

The research presents a hermeneutical phenomenological qualitative design, having as participants three mothers of two children and an adolescent – all three with severe disabilities, an 8-year-old girl with autism spectrum disorder (ASD), a 12-year-old girl with cerebral palsy (CP), and a 15-year-old adolescent with down syndrome (DS). The three parents agreed to voluntarily participate in the study, offering their testimonies about their role in educating their children with severe disabilities in the context of COVID-19, Peru. Children were diagnosed by professionals in hospitals belonging to the Ministry of Health, Peru, through a medical certificate that corroborates the disability of said children. However, some parents turn to other private hospital entities searching for a second diagnosis. This disability directly impacts the IQ of these children; before this, the Ministry of Education, Peru, considered three levels: mild, moderate, and severe. The mild and moderate conditions are attended in regular schools; the severe condition is attended in special schools where these children receive therapy services such as language, physical, psychological, and occupational to develop various skills interrupted by COVID-19. However, children have been receiving medical and educational care through social networks and virtual educational platforms in this context.

The planning of the study was developed based on three stages: 1. descriptive, 2. structural, and 3. discussion of results. Information was collected through a semi-structured interview with 26 questions distributed in four categories: 1. Being the mother of a child with a severe disability; 2. COVID-19 Pandemic; 3. Virtual education and 4. Future perspectives of families. All interviews were performed in the participants’ homes, applying the oriented biosafety protocols established by the WHO and endorsed by the Peruvian State [17]. The interview was validated by experts [18]. Theoretical information was extracted, mainly from scientific journals indexed in high-impact databases. In the descriptive stage, the research team designed the methodological and ethical deployment of the research. A previous informative talk was also planned to socialize the study’s importance, objectives, scope, educational relevance, and scientific relevance and explain the ethical protocols to the participants. In the structural stage, the functions of the researchers were delimited. In addition, methodological guidelines related to subject-object and logic-dialogic interactions were explained. In the discussion of results stage, a reflective analysis of the results obtained was carried out to code them according to the study categories. Finally, a private meeting was held with the mothers to confirm their testimonies so that they could also express coincidences or discrepancies with the interpretation made by the researchers. This step was the preamble to ensure the results and contrast them with previous studies. All the information obtained from the application of the in-depth interview was coded and then processed through the ATLAS.ti V9 program, allowing us to discover and analyze complex phenomena hidden in unstructured data.

## Results

**Figure 1.**
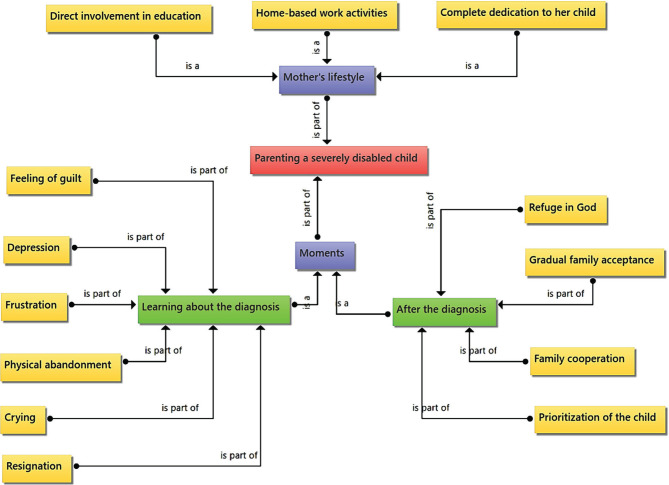
Category 1: Parenting a child with a severe disability.

It can be observed that mothers go through notable moments, the first, when they learn of their child’s diagnosis, accompanied by feelings of guilt, frustration, physical abandonment, crying, depression, and resignation, and the second moment after the diagnosis when it is common to seek refuge in religious questions, progressive acceptance of the family and prioritization of the child’s care (Figure 1).

**Figure 2.**
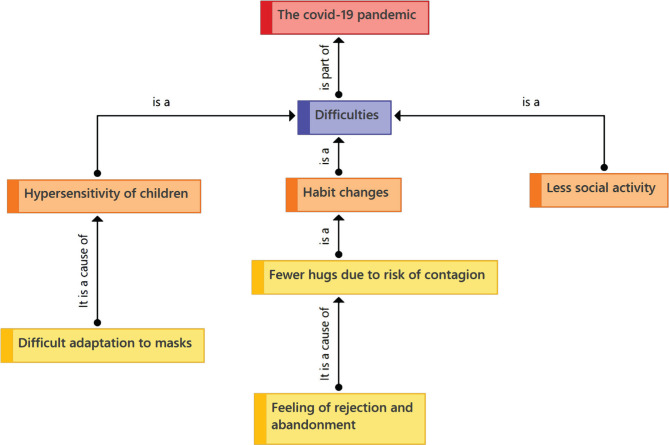
Category 2: The COVID-19 pandemic.

During the Covid-19 pandemic, mothers have reported serious difficulties coping with this scenario. The first difficulty reported was the adjustment to protocols such as masks given the hypersensitivity in the case of children with autism, for example. Another difficulty was the distancing and the decrease of some signs of affection, such as hugging, which generated a sense of rejection or abandonment (Figure 2).

**Figure 3.**
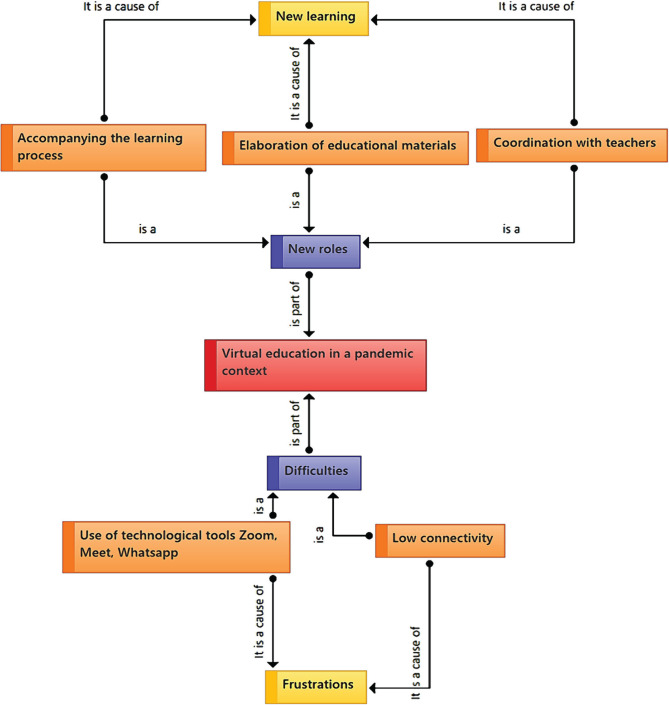
Category 3: Virtual education in the pandemic context.

Virtual education in pandemic contexts has generated difficulties for mothers of severely disabled children. These difficulties are related to technological tools and low connectivity, causing frustrations in them. Moreover, mothers report new roles assumed in this scenario: accompaniment in learning processes, coordination with teachers, and elaboration of educational materials (Figure 3).

**Figure 4.**
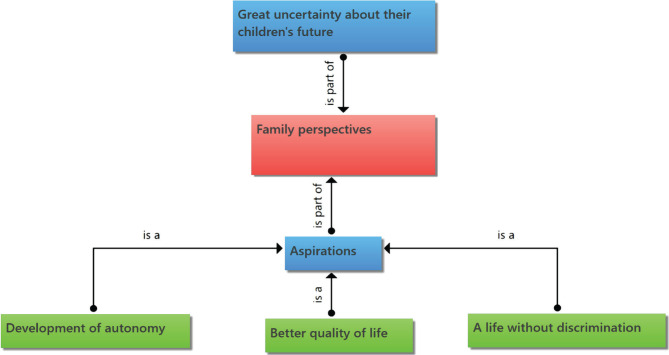
Category 4: Family perspectives.

Family perspectives can be classified into aspirations such as a better quality of life for their children, the search for autonomy, and a life without discrimination. On the other hand, mothers do not cease to consider the future as great uncertainty for their children (Figure 4).

## Discussion

### Being the mother of a child with a severe disability

The participants state that they experienced painful feelings upon learning of their children’s disability diagnosis. The mother of the girl with ASD says that her daughter’s autism diagnosis produced feelings of guilt and the death of her illusions of having an ideal daughter. She argues that her father went into depression. This process is complex for parents of children with disabilities because, like all parents, they idealize the birth of a perfect child. However, the reality is far from illusion, producing an emotional imbalance expressed through feelings of frustration and physical and emotional abandonment [19]. This painful experience led her to take refuge in God to appease her anguish. She says that after four months, the family began to change their attitude, accepting and becoming better informed about their daughter’s condition, so they assumed the need to prioritize the girl, providing her with the best living conditions and cooperating. Parents gradually accept their children’s disease during this challenging and complex process, making their initial feelings less acute. As they get used to the diagnosis and begin to provide the necessary care to their children, they also develop feelings of confidence, hope, and joy [20].

The mother of the adolescent with DS also presents a similar experience. She says that she had no idea that her son would have a disability since she had a quiet pregnancy, although the child was born prematurely. Upon teaching her about her son’s condition, she states that she did not affect him emotionally (at the interview, her bodily expression alerted more resignation than acceptance). This problematic situation worsened when the father resists accepting his son’s disability, expressing feelings of guilt and rejection, in addition to holding the mother responsible for it. For him, this disability was due to the mother’s age at her conception. Faced with this situation, she begins to sensitize the child’s father to accept reality, and little by little, she is achieving her purpose. This situation is not unfamiliar to other families with similar problems. However, over time the trend points to a kind of adaptation-resignation. The child needs his parents’ care to live in a balanced emotional climate to accept himself and recognize his uniqueness [19].

Upon learning of her daughter’s diagnosis during pregnancy, the mother of the girl with CP went into shock. She felt shocked because she was a desired daughter. She mentions that a recurring fear occurred in her that led her to uncertainty in the face of reality, triggering frequent crying. This change in her life causes her to find a medical solution to this painful experience that she was going through, ruling out abortion as an option. Likewise, she is committed to her family to educate the girl as much as possible and provide her with a good quality life. For many parents, the birth of a child with a disability means a revolutionary change at an integral level that allows them to objectively raise awareness of their reality and get involved in a new process of resignifying facts and facing this new challenge [21]. In general, the mother of the girl with ASD states that her son performs routine activities related to eating, dressing, playing, and schoolwork under her guidance and supervision. Also, he had to dedicate himself to the sale of food to help with household expenses; however, he acknowledges having received support from his family. The mother of the adolescent with DS refers to her family as nuclear. She shares her time between her domestic chores and work activity (sewing), which she performs from home, allowing her to dedicate herself to the development capabilities fully. The girl’s mother with CP assumes a leading role in developing her daughter’s skills; she fulfills the role of mother and partner. During the pandemic, families have changed their positions, providing emotional and social support to each member, facing a health crisis [22].

### COVID-19 pandemic

The mother of the girl with ASD kept her daughter confined for many months and taught her to use the mask, which was a complex process because people with autism are hypersensitive, which caused rejection and little tolerance. She resorted to visual aids techniques such as pictograms to achieve this learning. This experience is manifested in recent research that evaluates the gradual exposure and the molding to teach the use of the mask in children with ASD and the necessary and sufficient mechanisms for its continuous use, thus reducing the behavioral problems that originate from its learning [23]. Although there are no studies that indicate how children with ASD should use surgical masks, some recommend that these protective devices be sufficiently adaptable and ergonomic for the countenance of children; without neglecting the possible psychological impacts that the use of these masks may cause them [24].

The mother of the adolescent with DS mentions that initially, the quarantine caused her son a state of boredom because the child could not carry out routine leisure activities; she highlights that both she and the family had to change certain habitual affective behaviors; for example, they could not hug the child frequently. The adolescent’s understanding of this explanation was not immediate, although the adaptations began to be noticed as time passed. The mother fears that once COVID-19 is over, the process of returning to normality will be just as traumatic. Initially, this change generated in the adolescent feelings of rejection and abandonment from his parents, who made him understand that these measures were based on the love they had for him, and it was necessary to take protective measures to avoid contagion until it returned to normality. This situation is not accidental because the pandemic caused many families to stay at home and adapt to this new way of life, which led them to face difficult situations such as the tension between parents and children [25]. The adaptation process to a pandemic reality was complex for these mothers, who had to resort to various strategies to get their children to adapt to this new lifestyle related to health, nutrition, and family relationships. The mothers say that the confinement was brutal for their family, significantly altering the routine. They also say that, given the health condition of their children, they were very vulnerable to COVID-19 and feared for the lives of their children. Therefore, they needed to be vigilant to prevent any possibility of contagion. In this sense, many parents’ fear of exposing their children to COVID-19 caused them to take extreme and very radical measures, forcing them to permanently stay at home without social participation [25].

### Virtual education in the COVID-19 context

Mothers had to adapt to this new form of distance education, although they acknowledged that they were not prepared to take on their children’s education from home. For the mother of the girl with ASD, facing virtual tools such as Zoom and Meet, plus connectivity problems, generated nervousness, and frustration. However, she gradually adapted to it. At present, she recognizes the benefits of this teaching method by arguing that virtual education has developed in her daughter some skills that she did not expect of her. Recently developed research warns that although children with autism spectrum disorder are vulnerable to the effects of confinement, they have difficulties adapting to this new way of living and changes in their routines developed before the pandemic [26]. However, the mother feels that she saw her daughter advance, which relates to the commitment she assumed and her entire family. This achievement is also because the mother was looking to pave the way for the girl to be calm and carry out personal and school activities with pleasure. This made her feel like a successful mother. One study mentions that when methods to improve the behavior of children with ASD are used, and the results are seen, a feeling of success is felt in mothers, increasing hope and life satisfaction [27].

The adolescent’s mother with DS also had difficulties adapting to the new situation of virtual classes because she lacked the skills to handle information and communication technology (ICT) and follow the teacher’s guidance. This made her feel frustrated. However, she feels grateful for having received the support and motivation of the teacher to achieve favorable results in the learning of her son. He has substantially improved her memory, in addition to having increased her linguistic repertoire. Despite going through this complex adaptation process, she recognizes positive aspects of this experience, such as spending more time with the family, enjoying hobbies and leisure activities. This highlights the extraordinary capacity of mothers to face and accept the change of role in the education of their children, which is complex, taking into account the level of disability and the challenging behavior of the child in the pandemic context [28].

With CP, the girl’s mother states that this new experience was initially chaotic because she found a large gap between virtual and face-to-face education; firstly, it explained the difference between taking the girl to school, then picking her up, and then having the girl at home. It was hard to adapt; however, she achieved it by motivating and committing herself to her daughter’s learning. She created a space at home to record the evidence of learning that the teacher demanded. Conclusively, the mother considers that virtual education did not manage to develop expected learning in her daughter in contrast to what happened in face-to-face classes because the role of the teacher was assumed by her person, without having the professional preparation that this demands. Consequently, she had to learn specific music and tactile stimulation strategies, which helped her develop basic skills in this distance education. The study by Greenway *et al.* highlights that parents, despite having received guidance from schools and educational resources, express dissatisfaction when considering that these resources do not meet their children’s educational and psychological needs [29].

To develop communication skills and self-worth in their children, the mothers have worked together with the teachers, following the instructions, and applying them in experiential activities at home. The mother of the girl with ASD reports that her daughter performs routine activities such as washing, hanging, picking up, and putting away clothes; likewise, she has learned to control and clean her toilet, although she still needs supervision and support. It should be noted that people with ASD adhere to daily routines. However, isolation can change these routines, generating anxiety that influences their behavior; this occurs due to the interruption of educational and behavioral interventions necessary to develop positive skills [30]. To build language in her girl with ASD, the mother worked the anticipation technique and relied on pictograms, considering that she makes eye contact to achieve better results. She also resorted to reviewing some educational web pages to support making the materials with pictograms. It is necessary to consider that changes in the environment and routine can be complex for students with ASD. Therefore, it is essential to practice appropriate intervention mechanisms such as anticipation, which is used very early to produce significant results in behavioral treatment [31].

The adolescent’s mother with DS mentioned that the teacher guides the school tasks through video tutorials on WhatsApp, which the family puts into practice to develop daily life skills such as brushing teeth and going to the bathroom. For this task, they use images placed in strategic places where the activity is carried out; these activities are supervised by the teachers through the cell phone, providing timely feedback. However, she considers that it would have been better for the teacher to interact directly with her son to explain the activities to be carried out since she considers that she does not know the correct didactic methods as a mother. In the study by Deliyore *et al.*, the teachers who attend to students with intellectual disabilities in a virtual way did not manage to develop meaningful learning, despite the synchronous and collaborative interaction between teacher-student and parent [8].

In the case of the girl with CP, the mother said that the teacher sends the video tutorials that help her carry out the activities, highlighting as a priority the auditory-tactile sensory work due to suffering from cerebral palsy, for which she used images and sound objects, which allow the girl to hear and discriminate sounds because she has gradually lost her vision due to neurological problems, which even caused seizures. The girl depends on all family members and understands cleanliness, clothing, food etc. The needs of a family with a child with cerebral palsy are different according to the child’s functional capacity; if the disability is severe or highly dependent, the group requires resources and expensive medications since they present frequent medical problems, increasing the needs and financial limitations of families [32]. On the other hand, the mother considers the importance of physical therapy in this distance education, which must be carried out in a specialized center due to her daughter’s disability; however, this is not possible due to the girl’s vulnerability to the pandemic.

So that their children continue to be educated amid the pandemic, mothers have started developing educational materials for classes involving creativity, dedication, and time. In this regard, one of the mothers says that her son did not know that it was cardboard, plastic etc. In addition, they suggest that the state should invest in materials according to the educational needs of students with disabilities, whose distribution is regular and accessible, especially for families with low economic resources. Faced with this reality, mothers are aware of providing quality time in their children’s learning. However, they agree that they are not prepared to provide their children with an adequate education from their homes, leading them to high levels of stress, especially in those who have children with special educational needs [6].

### Prospects of families

Mothers’ expectations about the future of their children with disabilities are centered on developing their autonomy and a better quality of life within their possibilities. They want their children to be able to carry out some work activity without discrimination, considering that they will have to be autonomous at some point. Specifically, the girl’s mother with CP hopes that her daughter walks from her and can express a word by herself so that she has a better quality of life; without leaving aside the uncertainty of the future that awaits her daughter, and the case of helplessness. However, these aspirations are unlikely and harsh simultaneously, as the progression of cerebral palsy is uncertain and unpredictable. A recent study explains that although parents’ expectations of children with severe disabilities may be high, they are unclear due to fears, uncertainty, and lack of professional guidance to help families plan the future they want for their children [33].

Additionally, these expectations face a great barrier created by a society unable to tolerate and understand these people, with a lack of empathy and discrimination that lead to labeling them as “crazy”, “upset”, or “Mongoliths”; in the words of the mothers themselves, ignoring that these people like any other, deserve respect and consideration of the society where they live. Therefore, it is necessary to inform, raise awareness and sensitize society to eradicate all acts of discrimination against people with disabilities and thus learn to live in a just, equitable, tolerant, and inclusive community that respects the rights and opportunities of all.

## Conclusion

The participants acknowledge having gone through painful experiences in the face of their children’s disability diagnosis, developing feelings of guilt, uncertainty, depression, and denial about their child’s condition. This assimilation process was complex, leading them to take refuge in God and assume a double leading role of wife and mother to face this new challenge that involves caring for and raising a child with a disability. Similar and other complex situations have come to life for mothers in the current pandemic context. Mothers acknowledge that COVID-19 has changed the lifestyles of many families, including their own, feeling obliged to apply a set of biosafety protocols to safeguard family life, especially of their children with severe disabilities, who were more vulnerable to contracting the disease. However, this prolonged confinement helped strengthen their bonds, and they shared more time as a family. Mothers had to adapt virtual education to their children’s education from home, despite their limitations in ICT management, so they had to assume a new role that meant adding one more activity to their daily activities. In that sense, they reorganized the spaces and invested in technological equipment in connectivity to meet the new educational demands. Mothers show concern and uncertainty about the future that awaits their children with disabilities since they want to independently carry out some work activity. They also hope that their children can live in a more inclusive society with opportunities for all people.

## Acknowledgments

### Conflict of interest

The authors declare that there is no conflict of interest.

### Ethical approval

This study was approved by the Technical Committee for the review of doctoral program research projects (resolution No. 012 – 2020-DA-UCV).

### Consent to participate

Mothers voluntarily endorsed their participation in the research through an informed consent letter in which they defined their involvement. Confidentiality and anonymity were assured by the researchers who signed a letter of commitment to safeguard the identity of the participating mothers and use the information obtained from the in-depth interviews strictly for the research.

### Funding

This study was funded by the César Vallejo University, Peru.

### Authorship

PM, GC contributed to the conceptualization, investigation, writing, review and editing. EG, RA, contributed to data curation and validation. E-RN contributed to formal analysis, methodology and writing – review & editing. J-MV contributed to investigation and writing original draft. YJ-HF contributed to conceptualization and resources. MT contributed to validation and data curation.
